# A Network Pharmacology Approach to Uncover the Pharmacological Mechanism of XuanHuSuo Powder on Osteoarthritis

**DOI:** 10.1155/2016/3246946

**Published:** 2016-03-24

**Authors:** Huaqi Tang, Shuaibing He, Xinyue Zhang, Shilin Luo, Baixia Zhang, Xiaojie Duan, Zhiqian Zhang, Wenqi Wang, Yun Wang, Yikun Sun

**Affiliations:** School of Chinese Materia Medica, Beijing University of Chinese Medicine, Beijing 100102, China

## Abstract

As the most familiar type of arthritis and a chronic illness of the joints, Osteoarthritis (OA) affects a great number of people on the global scale. XuanHuSuo powder (XHSP), a conventional herbal formula from China, has been extensively applied in OA treatment. Nonetheless, its pharmacological mechanism has not been completely expounded. In this research, a network pharmacology approach has been chosen to study the pharmacological mechanism of XHSP on OA, and the pharmacology networks were established based on the relationship between four herbs found in XHSP, compound targets, and OA targets. The pathway enrichment analysis revealed that the significant bioprocess networks of XHSP on OA were regulation of inflammation, interleukin-1*β* (IL-1*β*) production and nitric oxide (NO) biosynthetic process, response to cytokine or estrogen stimuli, and antiapoptosis. These effects have not been reported previously. The comprehensive network pharmacology approach developed by our research has revealed, for the first time, a connection between four herbs found in XHSP, corresponding compound targets, and OA pathway systems that are conducive to expanding the clinical application of XHSP. The proposed network pharmacology approach could be a promising complementary method by which researchers might better evaluate multitarget or multicomponent drugs on a systematic level.

## 1. Introduction

Osteoarthritis (OA) refers to an illness of the joints and bones featured by progressive and chronic structural damage [1], particularly the destruction of articular cartilage, with joint instability, chronic pain, and stiffness as clinical symptoms [2]. Gender, age, obesity, traumatic injury, and metabolic dysfunction can be considered as the main OA risk factors [3]. Currently, most OA treatment strategies focus on reducing symptoms, enhancing function, and delaying time to surgery. There are three types of therapeutic agents, nonsteroidal anti-inflammatory drugs (NSAIDs), disease-modifying OA drugs (DMOADs), and biological and steroid response modifiers, which can relieve severity, reduce progression of disease, and avoid subsequent damage to the joints [4]. Although these strategies show excellent effects on OA, reports show that long-term application will cause side effects on the gastrointestinal tract [5].

As an important aspect of the complementary and alternative medical system, traditional Chinese medicine (TCM) has been widely utilized in the treating of OA for centuries [6] and has been proven efficient in relieving OA severity. In terms of the concept of TCM, OA can be classified as producing “arthromyodynia” (blockage syndrome, Bi syndrome, or Bi Zheng) [7]. However, application of TCM has been blocked by the absence of scientific comprehension regarding its mechanism. Therefore, it is important to explore and reveal the TCM mechanism.

The use of XuanHuSuo powder (XHSP) was recorded in* Pu Ji Fang,* which is regarded as the greatest herbal formula book in China and was written by Su Zhu in 1406 during the early Ming Dynasty. This formula is prepared from four Chinese herbs including* Angelica sinensis *(Oliv.) Diels (Dang Gui),* Corydalis yanhusuo *W. T. Wang (Yan Hu Suo),* Psoralea corylifolia *L. (Bu Gu Zhi), and* Achyranthes bidentata *Bl. (Niu Xi). Based on TCM theory, multiple agents in one formula should operate cooperatively. In terms of XHSP,* Angelica sinensis* and* Corydalis yanhusuo* are important for activating blood and strengthening analgesic functions;* Achyranthes bidentata* and* Psoralea corylifolia* can strengthen bone and tonify kidney;* Achyranthes bidentata* can emphasize actions of the formula on a particular region of the patient's body that suffers from OA or combine the action of other herbs in the formula. Recent studies show that compounds in each herb have curative efficacy for OA [8–12]; thus, the XHSP formula might be a novel therapeutic strategy for OA. However, the pharmacological mechanism has not been completely clarified.

Chinese herbal formulas (Fu-Fang) are multitarget and multicomponent agents that realize their particular therapeutic efficacy through regulation of the molecular network of body systems utilizing its active components [13]. Thus, new approaches and tactics are required to achieve systematic and comprehensive understanding of the herbal formula's mechanism. Hopkins has proposed the concept of network pharmacology [14] to investigate the influence or intervention of drugs and to show the synergism law of multicomponent drugs to seek high efficacy and low toxicity of multiple target medications. Meanwhile, the herbal formula is regarded as a multitarget and multicomponent therapeutic that meets the requirement of curing complicated illnesses in an integrated manner; thus, the methodology of network pharmacology can be used to pursue transcendental knowledge regarding the rules of combination in the formulas [15]. Therefore, a comprehensive network pharmacology method has been selected to understand the pharmacological mechanism of XHSP on OA, which offers a valuable opportunity for in-depth comprehension of the mechanism for inversing this illness-associated imbalanced network.

## 2. Materials and Methods

### 2.1. Data Preparation

#### 2.1.1. Composite Compounds of Each Herb in XHSP

Four hundred and thirty-five compounds from the four herbs found in XHSP were retrieved from references, TCM Database@Taiwan [16] (http://tcm.cmu.edu.tw/, updated in July 2013), which is the most comprehensive TCM database in the world, and the Traditional Chinese Medicine Systems Pharmacology Database [17] (TcmSP*™*, http://lsp.nwsuaf.edu.cn/, updated on May 31, 2014), a unique system pharmacology platform designed for Chinese herbal medicines. Thirty-two compounds in* Psoralea corylifolia*, 88 in* Corydalis yanhusuo*, 143 in* Angelica sinensis*, and 172 in* Achyranthes bidentata* were collected. The details are described in Table S1 (see Supplementary Material available online at http://dx.doi.org/10.1155/2016/3246946).

#### 2.1.2. Compound Targets for Each Herb in XHSP

All of the compound targets of each herb found in XHSP were collected from Stitch [18] (http://stitch.embl.de/, ver. 4.0) with the species limited as “*Homo sapiens*” and a confidence score >0.4. The compounds without relevant information were removed. Stitch is a resource to explore interactions between chemicals and proteins. Thus, we collected distinct targets related to the compounds in each herb found in XHSP. The details are described in Table S2.

#### 2.1.3. OA Targets

The OA targets were gathered from the therapeutic target database [19] (http://bidd.nus.edu.sg/group/ttd/ttd.asp, updated on September 15, 2013), which offers information about nucleic acid targets and therapeutic proteins. A total of 24 OA targets were gathered. The details are described in Table S3.

#### 2.1.4. Protein-Protein Interaction Data

Protein-protein interaction (PPI) data were a term from String [20] (http://string-db.org/, ver. 10), with the species limited to “*Homo sapiens*” and a confidence score >0.4, Database of Interacting Proteins [21] (DIP*™*, http://dip.doe-mbi.ucla.edu/dip/Main.cgi, updated on February 4, 2014), InAct [22] (http://www.ebi.ac.uk/intact/, ver. 4.2.1), and Molecular INTeraction database [23] (MINT, http://mint.bio.uniroma2.it/mint/Welcome.do, updated on August 30, 2011). Among them, String is a database of known and forecasted protein-protein interactions. DIP catalogs experimentally determined interactions between proteins and combines information from a variety of sources to create a single or consistent set of protein-protein interactions. InAct provides an open source database and analysis tools for molecular interaction data. And MINT focuses on experimentally verified protein-protein interactions mined from literature.

### 2.2. Network Construction

#### 2.2.1. Network Construction Method

Network construction was performed as follows: (1) a compound-compound target network was established by linking chemical compounds and corresponding targets [24]; (2) herb-compound target-OA target network was built by connecting the four XHSP herbs, corresponding compound targets, and OA targets that interacted with compound targets; and (3) compound target-OA target-other human proteins' PPI network was constructed by connecting compound targets, OA targets, and other human proteins that are directly interacting with the two targets.

All of the networks could be generated by utilizing the network visualization software Cytoscape [25] (http://cytoscape.org/, ver. 3.2.1), which is used to visualize biological pathways and networks of molecular interactions and to interact with these networks via profiles of gene expression, annotations, and other state data. The software then offers a basic set of features for data integration, analysis, and visualization for complicated network analysis.

#### 2.2.2. Network Topological Feature Set Definition

For every node in the network, three indices were applied to assess its topological features. “Degree” stands for the number of links or edges between a node and other nodes in a network [26]. “Node betweenness” evaluates the participation of a node in the shortest parts of a network and reflects the capability of nodes to manage the rate of information flow in the network [27]. “Closeness” is the inverse of the sum of the distance from node *i* to other nodes, which is a measurement of how long it will take to spread information from node *i* to other nodes [28]. The higher the degree, node betweenness, and closeness of a node are, the more significant it is in the network.

### 2.3. Pathway Enrichment Analysis

The Database for Annotation, Visualization and Integrated Discovery [29] (DAVID, https://david.ncifcrf.gov/home.jsp, ver. 6.7) was applied for Gene Ontology (GO) enrichment analysis.

## 3. Results and Discussion

### 3.1. Compound-Compound Target Network Analysis

**Figure 1:**
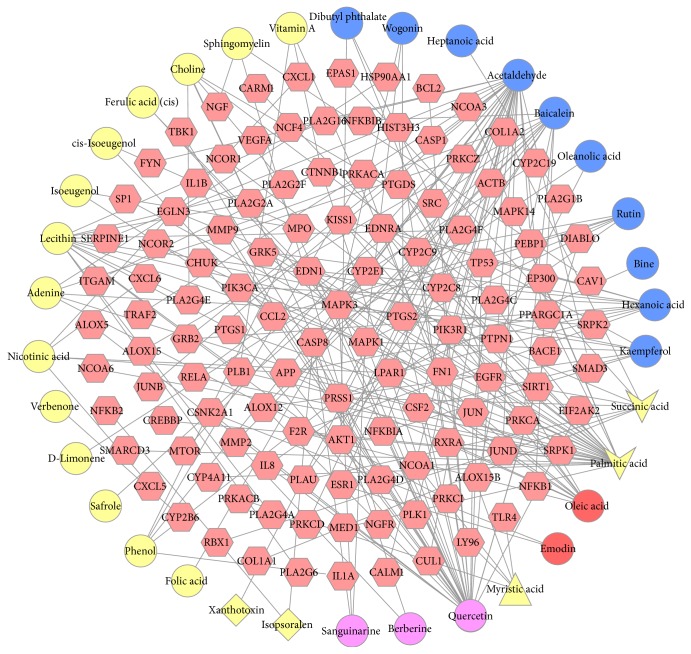
A compound-compound target network for XHSP on treating OA was built by linking 34 compounds and 122 compound targets. (Red hexagons represent compound targets. Yellow circles, blue circles, red circles, yellow diamonds, purple circles, yellow vee, and yellow triangle stand for the compounds contained in* Angelica sinensis*,* Achyranthes bidentata*,* Corydalis yanhusuo*,* Psoralea corylifolia*,* Corydalis yanhusuo *and* Achyranthes bidentata*,* Angelica sinensis *and* Achyranthes bidentata*, and* Psoralea corylifolia *and* Angelica sinensis *and* Achyranthes bidentata*, resp.)

The compound-compound target network is shown in Figure 1, including 156 nodes (34 compounds in XHSP yielded 122 compound targets) and 220 edges. In this network, targets in the interior circle show more interactions with compounds than those in the exterior. This indicates that a great number of compound targets can be regulated by multiple compounds instead of just one. For instance, EDN1 and PTGS2 are regulated by multiple ingredients including palmitic acid and quercetin. Besides, MAPK3, MAPK1, and CCL2 can also be regulated by more than one. Therefore, we could obtain an approximate observation of the relationship between compounds and compound targets from this network.

### 3.2. Herb-Compound Target-OA Target Network Analysis

**Figure 2:**
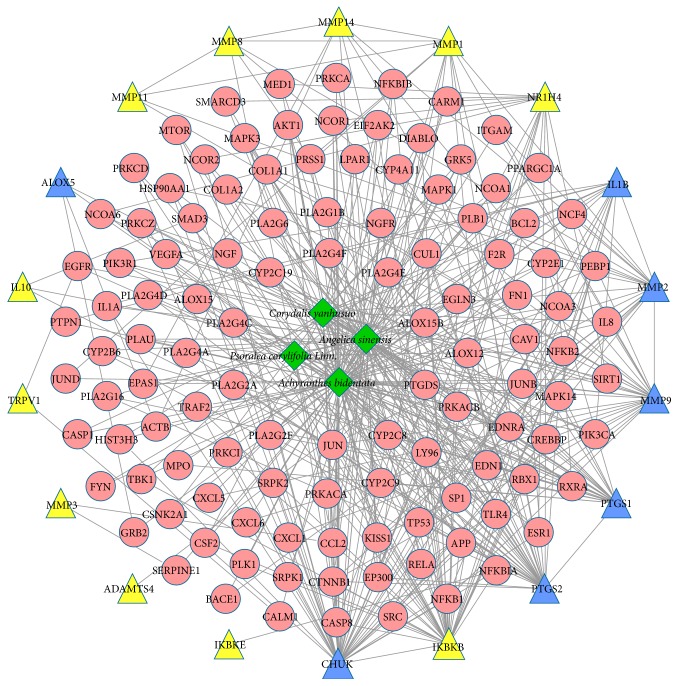
Herb-compound target-OA target network. (Green diamonds, red circles, yellow triangles, and blue triangles represent herbs, compound targets, OA targets, and compound targets/OA targets, resp.)

The herb-compound target-OA target network was constructed in order to identify the relationship between the four herbs in XHSP and the corresponding compound targets and OA targets. In Figure 2, the network was composed of 137 nodes (four herbs, 115 compound targets, 11 OA targets, and seven compound targets/OA targets) and 458 edges.* Achyranthes bidentata* showed the highest degree of distribution followed by* Angelica sinensis* and* Corydalis yanhusuo*, the links of which with other nodes were all over 40, thus, leading to their significance in the network.

**Figure 3:**
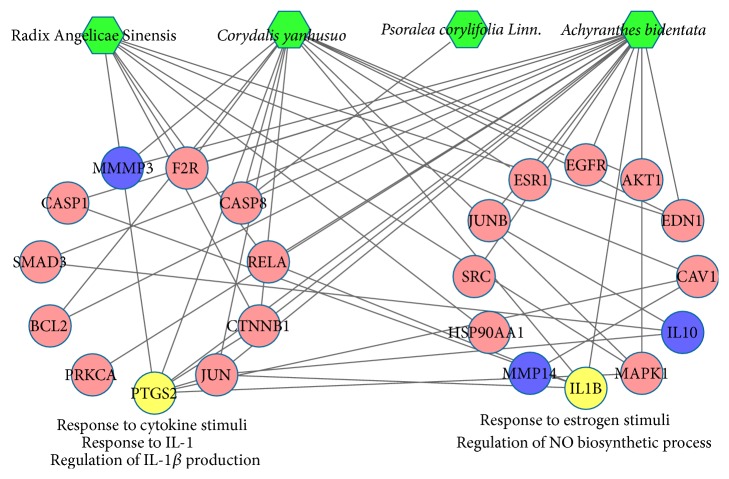
According to the associated biological processes or pathways, compound targets of XHSP and OA targets were related to various molecular mechanisms of OA. (Green nodes, pink circles, blue circles, and yellow circles represent four herbs in XHSP, compounds targets, OA targets, and compound targets/OA targets, resp.)

In Figure 3, according to the enrichment analysis, compound targets and OA targets were significantly associated with the regulation of nitric oxide (NO) biosynthetic process (GO ID: 45428, fold enrichment = 26.37, *P* < 0.001), response to interleukin-1 (IL-1) (GO ID: 70555, fold enrichment = 17.95, *P* = 0.012), regulation of interleukin-1*β* (IL-1*β*) production (GO ID: 32651, fold enrichment = 15.26, *P* = 0.016), response to cytokine stimuli (GO ID: 34097, fold enrichment = 12.88, *P* < 0.001), and response to estrogen stimuli (GO ID: 43627, fold enrichment = 8.72, *P* < 0.001). The details are described in Table S4.

The latest medical knowledge shows that the immune system is a key element in the progression of OA [30], and immune cytokines play a crucial role [31] by disturbing the anabolism and catabolism processes of tissue cells in joints [32]. The IL-1 family, which includes IL-1*α*, IL-1*β*, IL-1 receptor antagonist (IL-1Ra), and IL-18 [33], is significant in strengthening inflammatory reactions [34] and accelerating destruction of cartilage tissue [35]. This family of cytokines is especially involved in enhancing matrix metalloproteinases (MMPs) expression, which can speed up the synthesis and release of MMP zymogen, particularly MMPs 2, 3, 9, and 13, which increase matrix molecule degradation. IL-1 can also promote chondrocytes and synoviocytes to cause synovitis by producing prostaglandin E2 (PGE2) [36], which can reinforce the cartilage degradation effect of IL-1 and then accelerate OA progress. The increasing expression of IL-1 in OA synoviocytes can intensify the synovitis and the production of inflammatory cytokines and MMPs [37]. This is further confirmed by the existence of IL-1 in OA synovitis and degradation of the cartilage matrix [38].

According to reports, high levels of IL-1*β* can be found in the synovial membrane, synovial fluid, cartilage tissue, and subchondral bone tissue of OA patients [39]. This leads to a decrease in the synthesis of major extracellular matrix (ECM) components by inhibiting anabolic activity and increasing proteolytic enzyme yield in chondrocytes and subsequently degrading articular cartilage [40]. It also indicates that the degradation of cartilage in mice, rabbits, dogs, and pigs might be reduced by blocking IL-1*β* activity [41].

A number of proinflammatory factors, such as IL-1*β*, NO, and their downstream mediators, can activate the expression of MMPs [42], which particularly decrease the proteoglycans and native collagens [43]. For instance, NO is produced by inducible nitric oxide synthase (iNOS) [44]. Increasing levels of NO and iNOS could inhibit collagen and proteoglycan biosynthesis in OA patients [45], leading to the activation of MMPs and the inflammatory response [46]. Thus, NO shows the function of catabolism in OA cartilage.

Estrogen is closely associated with knee Osteoarthritis in middle-aged females [47]. According to recent research, females are more vulnerable to severe keen OA than males, especially after menopause [48]. Furthermore, the symptoms are higher in women than in men with the same level of radiographic damage. Thus, it could be hypothesized that the onset or progression of knee OA might relate to estrogen [49]. At the same time, other research found that estrogen deficiency may affect periarticular muscles, synovial lining, ligaments, articular capsule, and subchondral bone during the OA process [50]. All of this research indicates a potential protective role of estrogens in the OA process [51].

### 3.3. Compound Target-OA Target-Other Human Proteins' PPI Network Analysis

**Figure 4:**
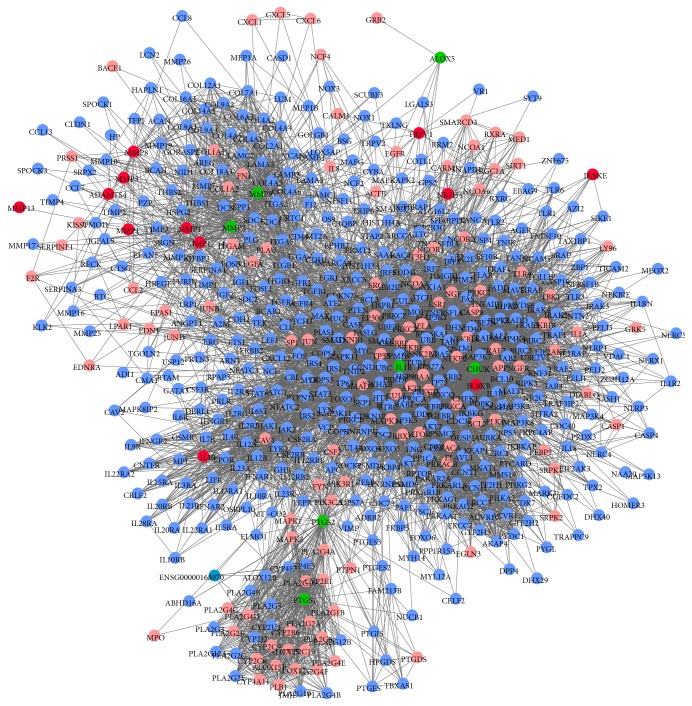
Compound target-OA target-other human proteins' PPI network. (Pink nodes, red nodes, green nodes, and blue nodes represent compound targets, OA targets, compound targets/OA targets, and other human proteins interacting with compound targets or OA targets, resp.)

To evaluate the significance of compound targets, a compound target-OA target-other human proteins' PPI network was constructed with 609 nodes (115 compound targets, 12 OA targets, seven compound targets/OA targets, and 475 other human proteins interacting with compound targets or OA targets) and 7379 edges (Figure 4). Three topological features of each node in the network were calculated to find the major nodes. Thus, 72 nodes with an average value of degree ≥24.19, node betweenness ≥0.00244, and closeness ≥0.4097 could be considered as major nodes (the details are described in Table S5). Finally, 41 major nodes, which intersected with compound targets, could be recognized as potential targets of XHSP.

**Figure 5:**
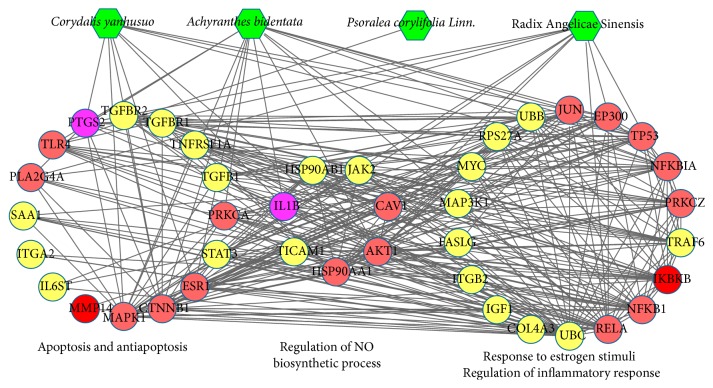
Direct interaction network between 41 major nodes in the XHSP compound target-OA target-other human proteins' PPI network. According to the correlative pathways, those nodes were divided into different functional modules, which were all related to OA. (Green diamonds, pink circles, red circles, purple circles, and yellow circles represent herbs in XHSP, compound targets, OA targets, compound targets/OA targets, and other human proteins interacting with compound targets or OA targets, resp.)

Furthermore, the direct interaction network between the 72 major nodes was established with 41 nodes (16 compound targets, four compound targets and/or OA targets, and 21 other human proteins interacting with compound targets or OA targets) and 318 edges. As shown in Figure 5, the major nodes could be categorized into various functional modules, including regulation of NO biosynthetic process (GO ID: 45428, fold enrichment = 28.23, *P* < 0.001), response to estrogen stimuli (GO ID: 43627, fold enrichment = 18.15, *P* < 0.001), antiapoptosis (GO ID: 6916, fold enrichment = 12.95, *P* < 0.001), regulation of inflammatory response (GO ID: 50727, fold enrichment = 10.03, *P* = 0.007), and apoptosis (GO ID: 6915, fold enrichment = 5.70, *P* < 0.001). The details are described in Table S6.

Research has shown that cartilage is sensitive to estrogen [52], a type of antiresorptive agent that can attenuate osteoclastic resorption and osteoclastogenesis [53]. Meanwhile, the beneficial effect of estrogen on OA prevention has been confirmed by animal experiment with estrogen replacement therapy (ERT) treatment [54]. In that study, fewer cartilage lesions were detected in ERT group than control group. In addition, experiments in rats [55], female rabbits [56], and monkeys [57] show that estrogen deprivation can increase bone turnover [58], accelerate cartilage breakdown [59], and increase bone resorption resulting in worsening of OA.

OA is characterized by inhibition of anabolic factor production and release of more catabolic factors [60], which leads to greater cartilage damage and a more severe inflammatory response [61]. Inflammatory cytokines play a crucial role [62] in disturbing the anabolism and catabolism of the joints [63]. Among these inflammatory cytokines, IL-1*β* is regarded as one of the most significant cytokines in OA pathogenesis [64]. IL-1*β* increases the expression of MMPs, which are crucial in the degradation of cartilage in OA [65], and induces the production of inflammatory mediators, which can result in clinical presentation of OA.

The treatment of OA is mainly based on the application of NSAIDs [66]; however, long-term application results in side effects on the gastrointestinal tract. In this regard, our data show that OA can be weakened by XHSP through reversing estrogen depletion and regulating inflammatory response by reducing the generation of inflammatory cytokines.

As two risk factors for OA, mechanical stress and inflammation are both related to NO upregulation [67]. NO is detrimental to the joint and makes contributions to the pathogenesis of OA [68] by modulating cytokine expression, suppressing matrix collagen and proteoglycan synthesis, activating MMPs, suppressing proliferation of chondrocytes, and promoting chondrocyte apoptosis [69]. NO manufactured by iNOS plays a complicated role in OA pathology and pain [70]. Utilizing iNOS or NO synthase inhibitors has resulted in great interest in therapeutic interventions, such as N^6^-(1-iminoethyl)-L-lysine hydrochloride [69] and S-methylisothiourea [71], which are effective in restraining OA progression. Therefore, the role of XHSP in the adjustment process of NO biosynthesis during OA should be an emphasis of further research.

Apoptosis, especially chondrocyte apoptosis, has been shown to have a close relationship with severity and progression of OA [72]. Chondrocytes are crucial in anabolic-catabolic balance for tissue function and matrix maintenance [73] and are capable of responding to structural changes in cartilage matrix [74]. Therefore, inhibition of chondrocyte apoptosis and maintenance of chondrocytes in a healthy condition might be a potential strategy against cartilage degeneration progression [75], preserving the complete cartilage and preventing its degeneration [76]. Current research has shown that berberine chloride is advantageous to matrix synthesis and cell survival in IL-1*β* stimulated chondrocytes and shows great therapeutic potential for cartilage repair in rat OA models [77]. Our study shows that XHSP directly interacts with chondrocyte apoptosis. Therefore, it can be hypothesized that XHSP acts via antiapoptosis activities to offer a protective effect on OA chondrocytes.

## 4. Conclusion

Perfect therapeutic strategies for OA have not been discovered yet, except for conventional strategies such as analgesics and NSAIDs. However, the side effects of long-term application of these treatments lead to increasing usage of TCM, which treats illnesses with various formulas to fit the patients' symptoms and constitutions. The TCM herbal formula is efficient in treating complicated illnesses such as OA due to its multicomponent and multitarget agent property, which could generate higher degrees of effectiveness with less toxic side effects. Unlike western medicine, TCM treats illness in a more systematic and holistic way. However, many studies are still applying the traditional research idea, “one-drug-one-target-one-illness,” which ignores the multitarget and multicomponent characteristic of TCM formula.

Due to the rapid development of bioinformatics, the network approach has become an innovative and effective means to uncover the molecular mechanisms underlying the curative effect of TCM formulas from a systematic viewpoint. In this research, we combined a number of network-based computational methods and algorithm-based approaches to predict targets, construct networks, and illuminate the molecular synergy of XHSP for OA. This provides clues to study the synergetic effects of each herb and pinpoint the major targets of XHSP and the corresponding molecular pathways on OA.

According to the prediction of network pharmacology, several signal pathways of XHSP acting on OA have been found for the first time. Regulation of inflammation, IL-1*β* production, NO biosynthetic processes, response to cytokines or estrogen stimuli, and antiapoptosis pathways were all identified as signaling pathways for the actions of XHSP on OA. All of these results are expected to help identify novel curative efficacy and take full clinical advantage of XHSP. In general, these results and network pharmacology approach could not only contribute to uncovering of the prescription rules of Chinese herb formula in clinical application but also demonstrate a holistic version of herbal remedies and facilitate the pharmacological evaluation of herb treatments. Based on our work, we believe that evaluating the efficacy of TCM formulas and uncovering the pharmacological mechanism on a systematic level will be a promising complementary and alternative method for future studies.

## Supplementary Material

Table S1 shows the composite compounds of each herb in XHSP. Table S2 shows the compound targets for each herb in XHSP. Table S3 shows the targets of osteoarthritis. Table S4 shows the enrichment analysis of compound target, OA target and compound target/OA target based on Gene Ontology (GO) annotation. Table S5 shows the topological features of major nodes in the compound target-OA target-other human proteins' PPI network. Table S6 shows enrichment analysis of compound target, OA target, compound target/OA target and other human protein based on Gene Ontology (GO) annotation.
